# The InBIO barcoding initiative database: DNA barcodes of Iberian Trichoptera, documenting biodiversity for freshwater biomonitoring in a Mediterranean hotspot

**DOI:** 10.3897/BDJ.11.e97484

**Published:** 2023-01-19

**Authors:** Joana Pauperio, Luis Martin Gonzalez, Jesus Martinez, Marcos A González, Filipa MS Martins, Joana Veríssimo, Pamela Puppo, Joana Pinto, Cátia Chaves, Catarina J. Pinho, José Manuel Grosso-Silva, Lorenzo Quaglietta, Teresa Luísa L Silva, Pedro Sousa, Paulo Celio Alves, Nuno Fonseca, Pedro Beja, Sónia Ferreira

**Affiliations:** 1 BIOPOLIS Program in Genomics, Biodiversity and Land Planning, CIBIO, Campus de Vairão, 4485-661 Vairão, Vila do Conde, Portugal BIOPOLIS Program in Genomics, Biodiversity and Land Planning, CIBIO, Campus de Vairão, 4485-661 Vairão Vila do Conde Portugal; 2 European Molecular Biology Laboratory, European Bioinformatics Institute, Hinxton, Cambridge, United Kingdom European Molecular Biology Laboratory, European Bioinformatics Institute Hinxton, Cambridge United Kingdom; 3 CIBIO, Centro de Investigação em Biodiversidade e Recursos Genéticos, InBIO Laboratório Associado, Campus de Vairão, Universidade do Porto, 4485-661 Vairão, Vila do Conde, Portugal CIBIO, Centro de Investigação em Biodiversidade e Recursos Genéticos, InBIO Laboratório Associado, Campus de Vairão, Universidade do Porto, 4485-661 Vairão Vila do Conde Portugal; 4 Departamento de Zoología, Genética y Antropología Física, Facultad de Biología. Universidad de Santiago de Compostela, Santiago de Compostela, Spain Departamento de Zoología, Genética y Antropología Física, Facultad de Biología. Universidad de Santiago de Compostela Santiago de Compostela Spain; 5 Departamento de Biologia, Faculdade de Ciências, Universidade do Porto, 4169-007, Porto, Portugal Departamento de Biologia, Faculdade de Ciências, Universidade do Porto, 4169-007 Porto Portugal; 6 Marshall University, Department of Biological Sciences, Huntington, United States of America Marshall University, Department of Biological Sciences Huntington United States of America; 7 Museu de História Natural e da Ciência da Universidade do Porto, Porto, Portugal Museu de História Natural e da Ciência da Universidade do Porto Porto Portugal; 8 CIBIO, Centro de Investigação em Biodiversidade e Recursos Genéticos, InBIO Laboratório Associado, Instituto Superior de Agronomia, Universidade de Lisboa, Lisboa, Portugal CIBIO, Centro de Investigação em Biodiversidade e Recursos Genéticos, InBIO Laboratório Associado, Instituto Superior de Agronomia, Universidade de Lisboa Lisboa Portugal; 9 EBM, Estação Biológica de Mértola, Praça Luís de Camões, Mértola, Portugal EBM, Estação Biológica de Mértola, Praça Luís de Camões Mértola Portugal

**Keywords:** Trichoptera, occurrence records, species distributions, continental Portugal, continental Spain, DNA barcode, cytochrome c oxidase subunit I (COI)

## Abstract

**Background:**

The Trichoptera are an important component of freshwater ecosystems. In the Iberian Peninsula, 380 taxa of caddisﬂies are known, with nearly 1/3 of the total species being endemic in the region. A reference collection of morphologically identified Trichoptera specimens, representing 142 Iberian taxa, was constructed. The InBIO Barcoding Initiative (IBI) Trichoptera 01 dataset contains records of 438 sequenced specimens. The species of this dataset correspond to about 37% of Iberian Trichoptera species diversity. Specimens were collected between 1975 and 2018 and are deposited in the IBI collection at the CIBIO (Research Center in Biodiversity and Genetic Resources, Portugal) or in the collection Marcos A. González at the University of Santiago de Compostela (Spain).

**New information:**

Twenty-nine species, from nine different families, were new additions to the Barcode of Life Data System (BOLD). A success identification rate of over 80% was achieved when comparing morphological identifications and DNA barcodes for the species analysed. This encouraging step advances incorporation of informed Environmental DNA tools in biomonitoring schemes, given the shortcomings of morphological identifications of larvae and adult Caddisﬂies in such studies. DNA barcoding was not successful in identifying species in six Trichoptera genera: *Hydropsyche* (Hydropsychidae), *Athripsodes* (Leptoceridae), *Wormaldia* (Philopotamidae), *Polycentropus* (Polycentropodidae) *Rhyacophila* (Rhyacophilidae) and *Sericostoma* (Sericostomatidae). The high levels of intraspecific genetic variability found, combined with a lack of a barcode gap and a challenging morphological identification, rendered these species as needing additional studies to resolve their taxonomy.

## Introduction

DNA barcoding is a molecular biology method for species identification that was proposed almost twenty years ago (Hebert et al. 2003). DNA barcoding relies on the comparison of a short mitochondrial DNA sequence of interest, usually a 658 bp fragment of the cytochrome c oxidase subunit I (COI) of the mitochondrial genome, known as the “Folmer region” (Folmer et al. 1994), although other regions and genes can also be used, including ones with different systematic scopes (e.g. Woese and Fox (1977)). For DNA barcoding to work, the sequence of interest must be compared to a library containing sequences with known species identification (Hebert et al. 2003, Hebert et al. 2004). As such, the construction of comprehensive reference libraries is essential and these require the morphological identification of vouchers by an expert taxonomist (Baird and Sweeney 2011, Ferreira et al. 2018, Kress et al. 2015). DNA barcoding applications have since expanded beyond single organism and species identification studies.

Development of DNA metabarcoding (Taberlet et al. 2012) was made possible with the advances in PCR technologies and high-throughput sequencing (HTS) (Liu et al. 2019). Multiple DNA barcodes are sequenced in a single sample, allowing the study of complex samples as bulk samples and environmental DNA. DNA metabarcoding has broadened the use of the two. DNA barcodes are now a ubiquitous tool in ecological and biological conservation studies, as well as, for example, in forensic applications (DeSalle and Goldstein 2019, Fišer Pečnikar and Buzan 2013, Kress et al. 2015).

Aquatic ecosystems are suffering high losses in biodiversity due to degradation and habitat destruction (Blancher et al. 2022). These ecosystems can be logistically challenging and time-consuming to monitor, as the current methodology is based on inventories and taxonomical diversity, based on morphology (Blancher et al. 2022). DNA metabarcoding has great potential for conservation and monitoring of aquatic ecosystems studies as it allows efficient, non-invasive and standardised sampling, without a priori knowledge of the existing biodiversity in an area (Thomsen and Willerslev 2015, Valentini et al. 2016). The choice of DNA markers and the biomass of the communities to monitor are important factors that can influence successful use of DNA metabarcoding (Thomsen and Willerslev 2015, Valentini et al. 2016, Casey et al. 2021).

The Trichoptera, or caddisﬂies, is an order of holometabolous insects that rank seventh overall amongst insect orders regarding species number, with 16,267 described species (Morse 2022) and is the most speciose of the primarily aquatic insect orders. Species of this order can be found in all continents, except Antarctica (Morse et al. 2019). While adults are mostly terrestrial and capable of flight, most species’ eggs, larvae and pupae are found in freshwater habitats (Morse et al. 2019). Adult caddisﬂies are moth-like insects having their bodies covered with setae or hairs (Holzenthal et al. 2007, Morse et al. 2019, Thomas et al. 2020). Their larvae are known for their ability to use silk to construct shelters and retreats, but some species can also be free-living (Casey et al. 2021, González and Cobo 2006, Holzenthal et al. 2015, Martín 2017, Martínez 2014, Morse et al. 2019, Thomas et al. 2020, Zhou et al. 2016). Caddisﬂy larvae provide several important ecological services, including their crucial role in the trophic dynamics and energy flow in the lakes, rivers and streams freshwater food webs (Holzenthal et al. 2015, Morse et al. 2019, Zhou et al. 2016). They show differential sensitivity to pollution and their diversity and abundance are widely used in biological freshwater monitoring (Resh and Rosenberg 1984). However, these programmes rely on larval morphological identification, which is much more challenging than adult determination and still impossible in the many species, whose larvae have not yet been described (Morse et al. 2019).

Environmental DNA has the potential to be used as a complement or as an alternative to the hurdles of current morphology-based identification in the scope of freshwater monitoring schemes (Lefrançois et al. 2020). However, successful application of eDNA in Europe will necessitate comprehensive reference collections of DNA sequences, representing existing European aquatic biodiversity (Baird and Sweeney 2011, Ferreira et al. 2018, Kress et al. 2015). Several studies have used barcodes to advance the knowledge on Trichoptera, either expanding the knowledge on their phylogeny or improving the DNA barcodes of Trichoptera species (e.g. Morinière et al. (2017), Zhou et al. (2016)).

In the Iberian Peninsula, approximately 380 Trichoptera taxa, from 23 families are known (Coppa et al. 2022, González et al. 1992, González and Martínez 2011, Malicky 2005, Martín 2017, Martínez 2014, Oláh et al. 2019a, Oláh et al. 2019b, Oláh et al. 2020, Valladolid et al. 2018, Titos et al. 2018). Of these, 374 are known in Spain and 190 in Portugal. The rate of endemicity of Iberian caddisﬂies is very high, with around one third of the taxa known to occur in the region being endemic (González et al. 1987, Martínez 2014, Martín 2017).

In this work, we present a contribution to the DNA barcode library of the Iberian Peninsula species of Trichoptera representing 37% (n = 142) of the Caddisflies known in the region and 38% (n = 57) of the known endemic Iberian taxa. This work was conducted within the framework of the InBIO Barcoding Initiative.

## General description

### Purpose

This dataset aims to provide a first contribution to an authoritative DNA barcode sequences library for Iberian Trichoptera, documenting biodiversity for freshwater biomonitoring in a Mediterranean hotspot. Such a library aims to enable DNA-based identification of species for both traditional molecular studies and DNA-metabarcoding studies. Furthermore, it constitutes a relevant resource for taxonomic research on Iberian Trichoptera and its distribution.

### Additional information

A total of 438 Trichoptera specimens were sequenced (Suppl. material 1). A full-length barcode of 658 bp was obtained for 400 specimens (91.3%) (Table 1, Suppl. material 2). These specimens represent 142 (37%) of the approximately 380 Caddisflies species known to occur in the Iberian Peninsula (González and Martínez 2011, Martínez 2014, Martín 2017). Furthermore, 57 taxa are Iberian endemics, representing 38% of the total endemic Iberian taxa (González and Martínez 2011, Martínez 2014, Martín 2017). The dataset includes 22 of the 23 families known to occur in the Iberian Peninsula (Table 1). These data contribute with 29 new taxa, 26 new species and three new subspecies of Trichoptera to the BOLD database (Table 1). For five additional species, the dataset contributes for the first time a full-length barcode.

Average nucleotide composition of the Trichoptera sequences is 37.7% thymine (T), 17.9% cytosine (C), 30.5% adenine (A) and 13.9% guanine (G), for a total GC content of 31.8% for the COI barcode fragment analysed. Genetic p-distances ranged from 0.00% between *Athripsodesalentexanus* Martín, González and Martínez, 2016 (n = 2) and *A.braueri* (Pictet, 1865) to 33.97% between *Ptilocolepusgranulatus* (Pictet, 1834) (n = 1) and *Potamophylaxlatipennis* (Curtis, 1834) (n = 4) (Suppl. material 3). Intraspecifc genetic p-distances ranged from 0.00% in 12 species, including several species of *Athripsodes*, *Hydropsyche* and *Rhyacophila* (average n = 3.16 specimens per species), to 6.16% in *Hydropsychepictetorum* Botosaneanu and Schmid, 1973 (n = 4), 6.22% in *Psychomyiapusilla* (Fabricius, 1781) (n = 7), 6.65% in *Rhyacophilamunda* McLachlan, 1862 (n = 9) and 7.45% in *Helicopsychelusitanica* McLachlan, 1884 (n = 2). Forty-seven species were represented by a single specimen in the dataset and, for this reason, no intraspecifc distance is calculated.

**Figure 1.**
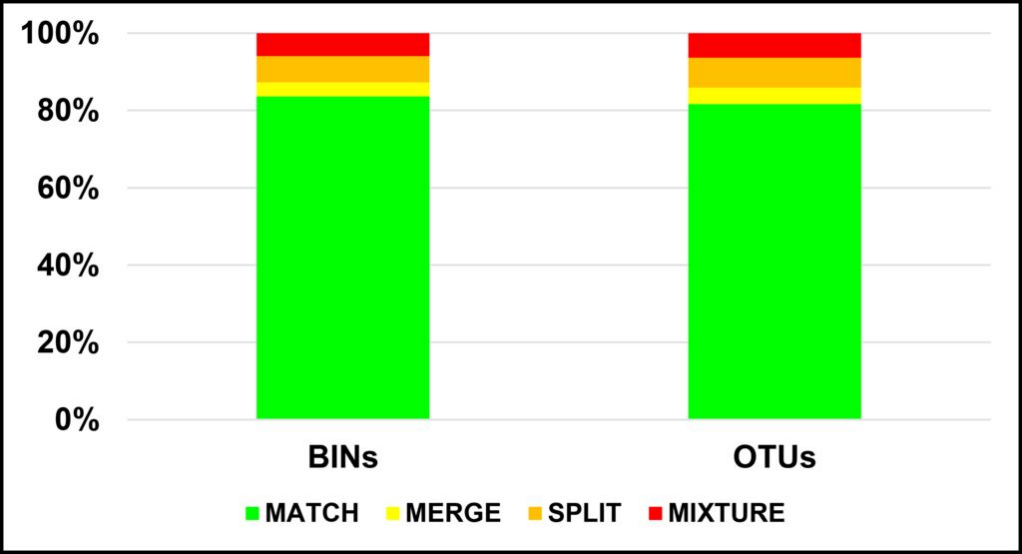
Comparison in OTU assignment performance between BOLD’s BIN and RESL stand-alone algorithms. The BIN dataset comprised 135 taxa (134 species) and the RESL stand-alone run comprised the entire 142 taxa (141 species) dataset. The four categories: MATCH, MERGE, SPLIT and MIXTURE into which the OTUs were divided, follow the criteria used by Ratnasingham and Hebert (2013).

The BOLD BIN system uses algorithms to cluster sequences into operational taxonomic units (OTUs) that closely correspond to species (Ratnasingham and Hebert 2013). A total of 146 BINs were retrieved by BOLD (Ratnasingham and Hebert 2007). Seven specimens have not been BIN attributed as their sequence is only 418 bp and no other specimens have been sequenced (Suppl. material 1). Two specimens, identified to the genus level only as *Helicopsyche* sp., clustered together in a separate BIN, “BOLD:AEC8747”. Of the 146 BINs, 45 BINs are unique to our dataset (Table 1, Suppl. material 1). Using the criteria followed by Ratnasingham and Hebert (2013), there were 83.6% of matches, 3.7% of merges, 6.7% of splits and 6.0% of mixtures when comparing BINs to the morphological identifications (Fig. 1). The BINs generated by BOLD clustered together sequences that closely agree with the morphological identifications of the specimens, with only a few exceptions in nine of the 22 Trichoptera families analysed.

The independent RESL run (Ratnasingham and Hebert 2013, Ratnasingham and Hebert 2007) retrieved 153 OTUs, plus one OTU for the *Helicopsyche* sp. specimens (Suppl. material 4). The differences found between the RESL OTUs and the morphological identifications were similar to those found between the latter and BOLD’s BINs, with 81.7% of matches, 4.2% of merges, 7.7% of splits and 6.3% of mixtures when comparing OTUs to the morphological identifications (Fig. 1).

Nevertheless, some differences existed between the RESL OTU clustering and the BINs created by BOLD (Suppl. materials 1, 4). In the family Hydropsychidae, sequences identified as *Hydropsycheinstabilis* (Curtis, 1834) clustered into a single OTU, but were split into two BINs. In the family Leptoceridae, sequences of specimens identified as *Athripsodesalentexanus* and *A.braueri* clustered in a single BIN. In the family Philopotamidae, sequences identified as *Philopotamusperversus* McLachlan, 1884 clustered into two OTUs, but were represented by a single BIN. In the family Polycentropodidae, sequences identified as *Polycentropusflavomaculatus* clustered into a single OTU, but were split into two BINs. In the family Rhyacophilidae, sequences identified as *R.dorsalis* (Curtis, 1834) and its subspecies, *R.d.albarracina* Malicky, 2002 clustered into a single OTU, but other sequences identified as *R.dorsalis* clustered into a different OTU. All *R.dorsalis* sequences share a single BIN, but the subspecies’ sequences have not been BIN attributed as their sequences are only 418 bp. Sequences identified as *R.intermedia* McLachlan, 1868 clustered into three OTUs, but were represented by a single BIN. Additionally, sequences identified as *R.martynovi* Mosely, 1930 clustered into two OTUs, but were represented by a single BIN. Furthermore, sequences identified as *Rhyacophilamunda* clustered into two OTUs, but were split into three BINs. In the family Sericostomatidae, there was no separation of the species *Sericostomapyrenaicum* and *S.vittatum*. These species clustered together into two different BINs, but sequences of *S.pyrenaicum* and *S.vittatum* also clustered in additional BINs (Suppl. materials 1, 4).

This work provided new DNA barcode sequences and distributional data for 436 specimens of Iberian Trichoptera, plus two French specimens. The dataset represents 37% of the Caddisflies known to occur in Iberia and the work added 29 taxa previously not represented in the BOLD database. To our knowledge, this is the first study to focus on DNA barcoding of the Trichoptera order for the Iberian Peninsula.

This study showed that DNA barcode sequences, based on the COI mitochondrial gene fragment, can be useful in identifying Iberian Trichoptera samples to species level. We achieved more than 80% success in matching the sequences generated to the morphological identification of the specimens. This is similar to the success rate achieved in 2017 (Morinière et al. 2017) for German Caddisﬂies (79.8%). A DNA barcode library is an essential tool for incorporating Environmental DNA techniques in monitoring schemes of aquatic ecosystems that use Iberian Caddisﬂies (Lefrançois et al. 2020). Our results constitute a first step in the construction of a DNA barcode database of a curated reference collection of Iberian Trichoptera species, which could be used to overcome the difficulties in identifying many of the Trichoptera larval specimens of traditional biological freshwater monitoring studies.

Incongruences were found in nine families. In six of them, Glossosomatidae, Helicopsychidae, Polycentropodidae, Limnephilidae, Rhyacophilidae and Psychomyiidae, the barcode analysis identified no species boundaries, with high levels of intraspecific genetic diversity (Suppl. material 3). It is possible that such levels of genetic diversity point to undescribed, distinct species. This hypothesis requires further morphological studies to search for diagnostic morphological traits that might separate these species.

In the family Hydropsychidae, nine species of the genus *Hydropsyche* could be identified through their barcodes and their genetic distances ranged between 13.4% and 23%. However, five other species could not be identified through DNA barcodes. These species, *H.ambigua*, *H.infernalis*, *H.pictetorum*, *H.siltalai* and *H.tenuis* were spliced between different BINs and OTUs, shared by some, but not all of the same species, further complicating their relationships. For the species with enough sequenced specimens, all were found to have moderate to high levels of intraspecific genetic diversity (Suppl. material 3). These species are difficult to identify morphologically and this study emphasises the need for further work towards a better understanding of the taxonomy of the genus in the Iberian Peninsula (Zamora-Muñoz et al. 2017).

In the family Leptoceridae, sequences identified as *Athripsodesalentexanus* and *A.braueri* clustered in a single BIN. All four sequences were identical. As such, DNA barcodes, based on COI, might not differentiate between these two species. This can be the result of an introgression event, if they had split very recently or alternatively, if their taxonomic identity needs revision.

In the family Philopotamidae, two *Wormaldiabeaumonti* and one *W.lusitanica* sequences were in the dataset. Two BINs are present in BOLD with both species represented in each (from previous data, but also with the new data). This genus is very difficult to identify morphologically and is likely that the morphological characters used are not able to separate both taxa.

In the family Sericostomatidae, there were problems separating two species of the genus *Sericostoma*, *S.pyrenaicum* and *S.vittatum*. These species clustered together into two different BINs, but sequences of *S.pyrenaicum* and *S.vittatum* also clustered in additional BINs. Intraspecific genetic diversity is relatively high in both species (2.49% and 2.89%, respectively). González et al. (1992) and Martínez (2014) already pointed out that, under these two names, a complex of species is actually hidden, some of them quite variable morphologically. A detailed morphological-molecular study may help to solve one of the most difficult taxonomic problems of our fauna. These findings suggest that both species need a taxonomic revision.

Our results did not corroborate the findings of Valladolid et al. (2018) and suggest further work is necessary regarding the identity of *Rhyacophilaadjuncta* and *R.sociata*. These authors restored the species *R.sociata*, previously considered a junior synonym of *R.denticulata* McLachlan, 1879. However, both BOLD clustering algorithms merged our samples, identified as *R.adjuncta* (2 specimens) and *R.sociata* (2 specimens), into a single BIN “BOLD:AAD5575”. Furthermore, this BIN includes all publicly available sequences in BOLD identified as *R.adjuncta* and *R.sociata*, including all sequences generated by Valladolid et al. (2018). In their paper, the authors did not investigate a possible relationship between these two species, nor was that relationship assessed in a subsequent study on the European species of the *R.fasciata* group (Valladolid et al. 2021). Finally, the BIN mentioned above also includes other sequences identified as *R.tristis* Pictet, 1834. and *R.fasciata* Hagen, 1859, although these are probably misidentifications.

We also identified several cases that require further study by taxonomists. Other possibilities for the incongruence found amongst the results include the existence of hybridisation, introgression or incomplete lineage sorting in these species, especially if they result from recent speciation events (e.g. Behrens-Chapuis et al. (2021), Morinière et al. (2017), Zhou et al. (2016)). These hypotheses require the combination of nuclear and mitochondrial markers to be resolved, preferably in an integrative taxonomic approach.

## Project description

### Title

The InBIO Barcoding Initiative Database: DNA Barcodes of Iberian Trichoptera 01

### Personnel

Luis Martín (taxonomist), Jesús Martínez (taxonomist), Marcos A. González (taxonomist), affiliated to Universidad de Santiago de Compostela; Pedro Beja (project coordinator), Joana Paupério (IBI manager), Sónia Ferreira (taxonomist and IBI manager), Filipa M.S. Martins (molecular biologist), Joana Veríssimo (molecular biologist), Pamela Puppo (molecular biologist), Joana C. Pinto (project technician), Cátia Chaves (project technician), Catarina J. Pinho (project technician), Pedro Sousa (project technician), Lorenzo Quaglietta (ecologist), Teresa Silva (molecular biologist), Paulo Célio Alves,(molecular biologist), Nuno Fonseca (bioinformatician), all affiliated to CIBIO-InBIO, University of Porto and José Manuel Grosso-Silva (taxonomist), affiliated to the MHNC-UP, University of Porto.

### Study area description

**Figure 2.**
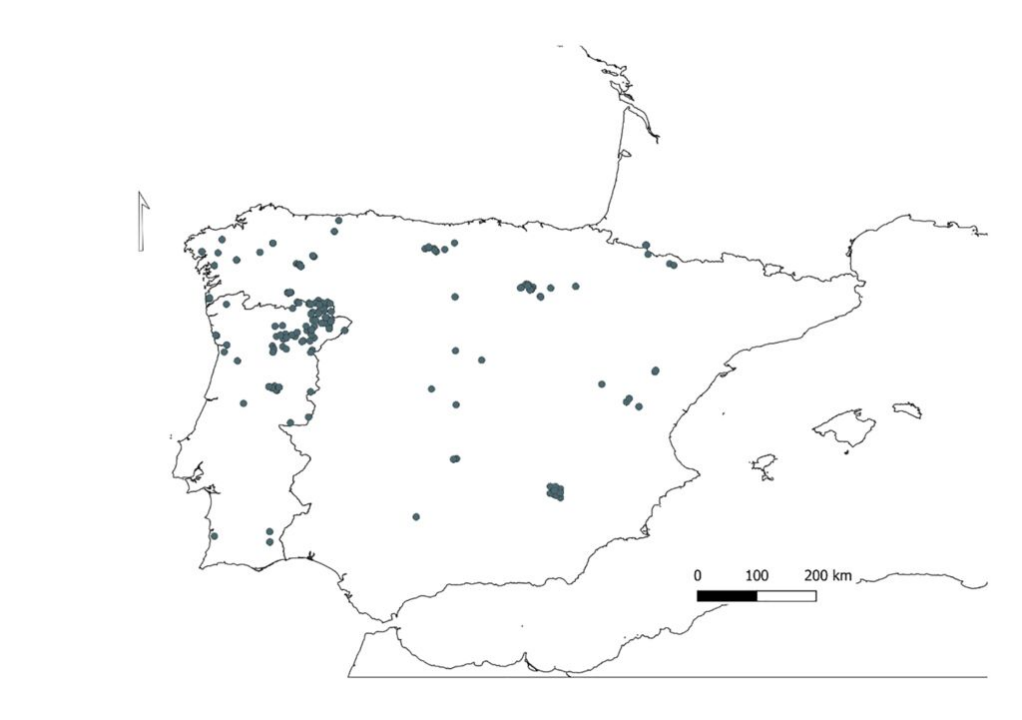
Sampling localities of the Trichoptera specimens analysed in this study. Nine localities could not be mapped because geographic coordinates were not available.

Iberian Peninsula (Fig. 2).

### Design description

Specimens were collected during field expeditions in the Iberian Peninsula, from 1975 to 2018 (n = 434 Fig. 2, Suppl. material 1), with more than 60% of specimens collected in the period between 2015 and 2017 (274 out of 434). Two additional specimens were collected in the French Pyrenees. Specimens kept at the InBIO Barcoding Initiative (IBI) reference collection (Vairão, Portugal), 230 in total, were stored in 96% ethanol. Specimens kept at the Colección Marcos A. González (Universidad de Santiago de Compostela, Spain), 206 in total, were stored in either 70% or 96% ethanol.

For each species, we selected, on average, three specimens for DNA sequencing, based on their location of capture, attempting to maximise the geographical coverage of the study.

DNA was extracted using two different kits: EasySpin Genomic DNA Microplate Tissue Kit (Citomed, Odivelas, Portugal) or QIAmp DNA Micro Kit (Qiagen, Hilden, Germany). QIAmp DNA Micro Kit is designed to extract higher concentrations of genetic material from samples with small amounts of DNA.

DNA amplification was performed using three different primer pairs, that amplify three overlapping fragments of the same 658 bp region of the COI mitochondrial gene. In the beginning of the project (2015), we used two primer pairs, LCO1490 (Folmer et al. 1994) + Ill_C_R (Shokralla et al. 2015) and Ill_B_F (Shokralla et al. 2015) + HCO2198 (Folmer et al. 1994) (henceforth referred to as LC and BH, respectively) to amplify two overlapping fragments of 325 bp and 418 bp, respectively. After the publication of the third primer pair, BF2 + BR2 (422 bp fragment), by (Elbrecht and Leese 2017), this started to be used instead of the second primer pair (Ill_B_F + HCO2198) due to higher amplification efficiency. PCRs were performed in 10 µl reactions, containing 5 µl of Multiplex PCR Master Mix (Qiagen, Germany), 0.3 (BF2-BR2) – 0.4 mM of each primer, and 1-2 µl of DNA, with the remaining volume in water. The thermocycling for PCR reactions was performed in T100 Thermal Cycler (Bio-Rad, California, USA) and carried out with an initial denaturation at 95ºC for 15 min, followed by 5 cycles at 95ºC for 30 sec, 47ºC for 45 sec, 72ºC for 45 sec (only for LC and BH); then 40 cycles at 95ºC for 30 sec, 51ºC for 45 sec (48ºC for 60 sec for BF2 + BR2), 72ºC for 45 sec; and a final elongation step at 60ºC for 10 min.

All PCR products were analysed by agarose gel electrophoresis and samples selected for sequencing were then organised for assignment of sequencing ‘indexes’. One of two types of index was used for each run. For Illumina indexes, samples were pooled into one plate, as described in Shokralla et al. (2015). When using custom indexes, designed, based on Meyer and Kircher (2010), no pooling was required. The latter allow for a maximum of 1920 unique index combinations. A second PCR was then performed where the ‘indexes’ and Illumina sequencing adapters were attached to the PCR product. The index PCR was performed in a volume of 10 µl, including 5 µl of Phusion® High-Fidelity PCR Kit (New England Biolabs, U.S.A.) or KAPA HiFi PCR Kit (KAPA Biosystems, U.S.A.), 0.5 µl of each ‘index’ and 2 µl of diluted PCR product (usually 1:4). This PCR reaction runs for 10 cycles at an annealing temperature of 55ºC. The amplicons were purified using AMPure XP beads (Beckman Coulter Genomics, Massachusetts, United States) before quantification using NanoDrop 1000 (Thermo Fisher Scientific, Massachusetts, USA). Concentrations between samples were then normalised and samples were pooled, based on used primer sets. Quantification of final pools was assessed through qPCR using the KAPA Library Quantification Kit Illumina® Platforms (Kapa Biosystems) and the 2200 Tapestation System (Agilent Technologies, California, USA) was used for fragment length analysis as described by Paupério et al. (2018).

Sequencing was conducted in an Illumina MiSeq benchtop system, using a V2 MiSeq sequencing kit (2x 250 bp) to perform sequencing at CIBIO facilities.

Sequences were filtered and processed with OBITools (Boyer et al. 2015) and the fragments were assembled into their consensus 658 bp-long sequences using Geneious 6.1.8 (https://www.geneious.com). The obtained DNA sequences were then compared against the Barcode of Life Data Systems (BOLD) database (Ratnasingham and Hebert 2007) using the built-in identification engine, based on the BLAST algorithm. Sequences were submitted to the BOLD database and the Barcode Index Numbers (BIN) for every sequence were retrieved and analysed (Suppl. materials 1, 2). As not all our sequences matched the criteria used in BOLD (sequence length) to be clustered in a BIN, we ran the Refined Single Linkage algorithm (RESL, Ratnasingham and Hebert (2013)) on our dataset in the BOLD system (Ratnasingham and Hebert 2007) in an independent run (Suppl. material 4). This process clusters sequences independent of their BIN registry, generating OTUs that can be analysed independently.

All DNA barcode sequences were aligned in Geneious 6.1.8 with MUSCLE (Edgar 2004) plug-in. Nucleotide composition of all sequences, as well as intra and interspecific p-distances,were calculated in MEGA11 (Tamura et al. 2021).

### Funding

InBIO Barcoding Initiative is funded by the European Union’s Horizon 2020 Research and Innovation Programme under grant agreement No 668981 and by the project PORBIOTA — Portuguese E-Infrastructure for Information and Research on Biodiversity (POCI-01-0145-FEDER-022127), supported by Operational Thematic Program for Competitiveness and Internationalization (POCI), under the PORTUGAL 2020 Partnership Agreement, through the European Regional Development Fund (FEDER). The fieldwork benefited from EDP Biodiversity Chair, including research conducted at the Long Term Research Site of Baixo Sabor (LTER_EU_PT_002), the project “Promoção dos serviços de ecossistemas no Parque Natural Regional do Vale do Tua: Controlo de Pragas Agrícolas e Florestais por Morcegos” funded by the Agência de Desenvolvimento Regional do Vale do Tua. SF was supported by individual research contract (2020.03526.CEECIND) and CJP, JV and FMSM by a PhD grant (SFRH/BD/145851/2019; SFRH/BD/133159/2017; SFRH/BD/104703/2014) funded by FCT.

## Sampling methods

### Study extent

Iberian Peninsula.

### Sampling description

Specimens were captured during direct searches of the environment, using mainly hand-held sweep-nets or lured by light trapping, the latter with UV (black-light) LEDs. Morphological identification was done, based on Malicky (2004) using a stereoscopic microscope for the study of genitalia. In some cases, genitalia were cleared in 10% potassium hydroxide (KOH) at room temperature for 4–8 hours, rinsed in water and placed in a drop of glycerine or resin (DMHF) on a clean slide for further study. From each specimen, one tissue sample (a leg) was removed and stored in 96% ethanol for DNA extraction at the IBI collection.

### Quality control

All DNA barcode sequences were compared against the BOLD database and the 99 top results were inspected in order to detect possible problems due to contaminations or misidentifications. Prior to GBIF submission, data were checked for errors and inconsistencies with OpenRefine 3.3 (http://openrefine.org).

### Step description

Specimens were collected in 66 different localities in Portugal and 74 localities in Spain. Collections were carried out between 1975 and 2018. Specimens were collected during fieldwork by direct search of specimens, by sweeping the vegetation with a hand-net and by using light traps and were preserved in 96% alcohol. Captured specimens were deposited in the IBI reference collection at CIBIO (Research Center in Biodiversity and Genetic Resources) or in the collection Marcos A. González at the University of Santiago de Compostela (Spain). Specimens were morphologically identified with the assistance of stereoscopic microscopes (Leica MZ12, 8x to 100x; Olympus SZX16, 7x to 115x). DNA barcodes were sequenced from all specimens. For this, one leg was removed from each individual, DNA was then extracted and a 658 bp COI DNA barcode fragment was amplified and sequenced. All obtained sequences were submitted to BOLD and GenBank databases and, to each sequenced specimen, the morphological identification, when available, was contrasted with the results of the BLAST of the newly-generated DNA barcodes in the BOLD Identification Engine. Prior to submission to GBIF, data were checked for errors and inconsistencies with OpenRefine 3.3 (http://openrefine.org/).

## Geographic coverage

### Description

Specimens were collected in the Iberian Peninsula, 229 from 66 localities in Portugal and 207 from 74 localities in Spain (Fig. 2, Suppl. material 5 for further details). Two additional specimens were collected in two French localities. The *Rhyacophilalaevis* Pictet, 1834 specimen represented in the dataset was collected in the French Pyrenees.

### Coordinates

-8.94 and -0.22 Latitude; 42.89 and 37.50 Longitude.

## Taxonomic coverage

### Description

This dataset is composed of data relating to 438 Trichoptera specimens. All specimens were determined to species level, with 14 specimens further identifed to subspecies level. Overall, 141 species are represented in the dataset. These species belong to 22 families.

### Taxa included

**Table:** 

Rank	Scientific Name	Common Name
kingdom	Animalia	Animals
subkingdom	Eumetazoa	
phylum	Arthropoda	
class	Insecta	
family	Apataniidae	
family	Beraeidae	
family	Brachycentridae	
family	Calamoceratidae	
family	Ecnomidae	
family	Glossosomatidae	
family	Goeridae	
family	Helicopsychidae	
family	Hydropsychidae	
family	Hydroptilidae	
family	Lepidostomatidae	
family	Leptoceridae	
family	Limnephilidae	
family	Odontoceridae	
family	Philopotamidae	
family	Phryganeidae	
family	Polycentropodidae	
family	Psychomyiidae	
family	Ptilocolepidae	
family	Rhyacophilidae	
family	Sericostomatidae	
family	Uenoidae	

## Temporal coverage

**Data range:** 1975-5-03 – 2018-5-16.

## Collection data

### Collection name

InBIO Barcoding Initiative

### Collection identifier

4ec2b246-f5fa-4b90-9a8d-ddafc2a3f970

### Specimen preservation method

“Alcohol”

### Curatorial unit

DNA extractions - 1 to 438

## Usage licence

### Usage licence

Creative Commons Public Domain Waiver (CC-Zero)

## Data resources

### Data package title

The InBIO Barcoding Initiative Database: DNA Barcodes of Iberian Trichoptera

### Resource link

http://dx.doi.org/10.5883/DS-IBITR01

### Number of data sets

1

### Data set 1.

#### Data set name

DS-IBITR01 IBI-Trichoptera 01

#### Data format

dwc, xml, tsv, fasta

#### Download URL

http://www.boldsystems.org/index.php/Public_SearchTerms?query=DS-IBITR01

#### Description

The InBIO Barcoding Initiative Database: DNA Barcodes of Iberian Trichoptera dataset can be downloaded from the PublicData Portal of BOLD (http://www.boldsystems.org/index.php/Public_SearchTerms?query=DS-IBITR01) in different formats (data as dwc, xml or tsv and sequences as fasta files). Alternatively, BOLD users can log-in and access the dataset via the Workbench platform of BOLD. All records are also searchable within BOLD, using the research function of the database. The InBIO Barcoding Initiative will continue sequencing Iberian Trichoptera for the BOLD database, with the ultimate goal of comprehensive coverage. The version of the dataset, at the time of writing the manuscript, is included as in the form of one text file for record information as downloaded from BOLD, one text file with the collection and identification data in Darwin Core Standard format (downloaded from GBIF, Martín et al. (2022)) and of a fasta file containing all sequences as downloaded from BOLD. It should be noted that, as the BOLD database is not compliant with the Darwin Core Standard format, the Darwin Core formatted file (dwc) that can be downloaded from BOLD is not strictly Darwin Core formatted. For a proper Darwin Core formatted file, see http://ipt.gbif.pt/ipt/resource?r=ibi_trichoptera_01&amp; v = 1.1 (Suppl. material 5). All data are available in the BioStudies database (http://www.ebi.ac.uk/biostudies) under accession number S-BSST920.

**Data set 1.** 

Column label	Column description
processid	Unique identifier for the sample.
sampleid	Identifier for the sample being sequenced, i.e. IBI catalogue number at Cibio-InBIO, Porto University. Often identical to the "Field ID" or "Museum ID".
recordID	Identifier for specimen assigned in the field.
catalognum	Catalogue number.
fieldnum	Field number.
institution_storing	The full name of the institution that has physical possession of the voucher specimen.
bin_uri	Barcode Index Number system identifier.
phylum_taxID	Phylum taxonomic numeric code.
phylum_name	Phylum name.
class_taxID	Class taxonomic numeric code.
class_name	Class name.
order_taxID	Order taxonomic numeric code.
order_name	Order name.
family_taxID	Family taxonomic numeric code.
family_name	Family name.
subfamily_taxID	Subfamily taxonomic numeric code.
subfamily_name	Subfamily name.
genus_taxID	Genus taxonomic numeric code.
genus_name	Genus name.
species_taxID	Species taxonomic numeric code.
species_name	Species name.
identification_provided_by	Full name of primary individual who assigned the specimen to a taxonomic group.
identification_method	The method used to identify the specimen.
voucher_status	Status of the specimen in an accessioning process (BOLD controlled vocabulary).
tissue_type	A brief description of the type of tissue or material analysed.
collectors	The full or abbreviated names of the individuals or team responsible for collecting the sample in the field.
lifestage	The age class or life stage of the specimen at the time of sampling.
sex	The sex of the specimen.
lat	The geographical latitude (in decimal degrees) of the geographic centre of a location.
lon	The geographical longitude (in decimal degrees) of the geographic centre of a location.
elev	Elevation of sampling site (in metres above sea level).
country	The full, unabbreviated name of the country where the organism was collected.
province_state	The full, unabbreviated name of the province ("Distrito" in Portugal) where the organism was collected.
region	The full, unabbreviated name of the municipality ("Concelho" in Portugal) where the organism was collected.
exactsite	Additional name/text description regarding the exact location of the collection site relative to a geographic relevant landmark.

## Supplementary Material

1FEDCDFE-9607-52E2-9281-9A2397F8BAB7 10.3897/BDJ.11.e97484.suppl1 Supplementary material 1 IBI-Trichoptera 01 library - Specimen details Data type Record information - specimen data Brief description The file includes information about all records in BOLD for the IBI-Trichoptera 01 library. It contains collection and identification data. The data are as downloaded from BOLD, without further processing. File: oo_767197.txt https://binary.pensoft.net/file/767197 Joana Paupério, Luis Martín, Sónia Ferreira, Jesús Martínez, Marcus A. González, Martin Corley, José Manuel Grosso-Silva, Lorenzo Quaglietta, Pedro Sousa, Pedro Beja

BD36592B-C812-56E8-AE84-C7B430DBD383 10.3897/BDJ.11.e97484.suppl2 Supplementary material 2 IBI-Trichoptera 01 library - DNA sequences Data type Genomic data, DNA sequences Brief description COI sequences in fasta format. Each sequence is identified by the BOLD ProcessID, species name, marker and GenBank accession number, separated by pipe. The data are as downloaded from BOLD. File: oo_767198.fas https://binary.pensoft.net/file/767198 Joana Paupério, Luis Martín, Sónia Ferreira, Jesús Martínez, Marcus A. González, Filipa M.S. Martins, Joana Veríssimo, Pamela Puppo, Joana C. Pinto, Cátia Chaves, Catarina Pinho, Pedro Sousa, Pedro Beja

F61B92AF-FE1F-5021-ADFA-096098EEB7BE 10.3897/BDJ.11.e97484.suppl3 Supplementary material 3 Genetic Distances Data type Genetic distances between analysed specimens Brief description Brief description: Estimates of average genetic divergence (uncorrected p- distances) for species of Trichoptera. Values under the diagonal refer to interspecifc divergence, while values in the diagonal represent intraspecifc divergence. File: oo_760686.xlsx https://binary.pensoft.net/file/760686 Joana Paupério, Luis Martín, Sónia Ferreira, Jesús Martínez, Marcus A. González, Filipa M.S. Martins, Joana Veríssimo, Pamela Puppo, Joana C. Pinto, Cátia Chaves, Catarina Pinho, José Manuel Grosso-Silva, Lorenzo Quaglietta, Pedro Sousa, Paulo Célio Alves, Nuno Fonseca, Pedro Beja

75803079-CCD9-5FD2-AD5F-9E0FA01A5FA2 10.3897/BDJ.11.e97484.suppl4 Supplementary material 4 OTUs generated by the Refined Single Linkage algorithm (RESL,) Data type OTUs generated by the RESL algorithm and respective sequence composition Brief description OTUs generated by the RESL algorithm (Ratnasingham and Hebert, 2013) in the BOLD system (Ratnasingham and Hebert, 2007), respective sequence composition and Nearest Neighbour genetic distance. The data are downloaded from BOLD, without further processing. File: oo_760687.xlsx https://binary.pensoft.net/file/760687 Joana Paupério, Luis Martín, Sónia Ferreira, Jesús Martínez, Marcus A. González, Filipa M.S. Martins, Joana Veríssimo, Pamela Puppo, Joana C. Pinto, Cátia Chaves, Catarina Pinho, José Manuel Grosso-Silva, Lorenzo Quaglietta, Pedro Sousa, Paulo Célio Alves, Nuno Fonseca, Pedro Beja

A87E8E8D-6F1A-54C7-AEF5-46129EE5F371 10.3897/BDJ.11.e97484.suppl5 Supplementary material 5 IBI-Trichoptera 01 library - Specimen details - Darwin Core Standard Data type Record information - specimen data in Darwin Core Standard format Brief description The file includes information about all records in BOLD for the IBI-Trichoptera 01 library. It contains collection and identification data. The data are downloaded from GBIF, without further processing. File: oo_760685.txt https://binary.pensoft.net/file/760685 Luis Martín, Sónia Ferreira, Jesús Martínez, Marcus A. González, Martin Corley, José Manuel Grosso-Silva, Lorenzo Quaglietta, Pedro Sousa, Pedro Beja

## Figures and Tables

**Table 1.** List of species that were collected and DNA barcoded within this project.

**Family**	**Taxa**	**IBI code**	**BOLD code**	**BOLD BIN**
Apataniidae	*Apataniatheischingerorum* Malicky, 1981	INV05962	IBITR421-20	BOLD:ADL7734
Beraeidae	*Beraeaalva* Malicky, 1975	INV05488	IBITR348-20	BOLD:AAJ8091
	*Beraeamalatebrera* Schmid, 1952	INV04753	IBITR267-20	BOLD:AAO2491
		INV04267	IBITR173-20	
Brachycentridae	*Micrasemacenerentola* Schmid, 1952	INV05952	IBITR414-20	BOLD:AAO3157
	*Micrasemalongulum* McLachlan, 1876	INV06484	IBITR435-20	BOLD:AAK7456
	*Micrasemaminimum* McLachlan, 1876	INV05973	IBITR430-20	BOLD:AAH6898
		INV05974	IBITR431-20	
	*Micrasemamoestum* (Hagen, 1868)	INV00475	IBITR054-20	BOLD:AAO1660
		INV00476	IBITR055-20	
		INV00477	IBITR056-20	
	*Micrasemaservatum* (Navás, 1918)	INV04731	IBITR252-20	BOLD:AAH3018
		INV04732	IBITR253-20	
Calamoceratidae	*Calamocerasmarsupus* Brauer, 1865	INV02470	IBITR101-20	BOLD:AAO2482
		INV05476	IBITR336-20	
Ecnomidae	*Ecnomusdeceptor* McLachlan, 1884	INV03539	IBITR140-20	BOLD:ABU6618
		INV03546	IBITR141-20	
		INV03605	IBITR146-20	
		INV05502	IBITR360-20	
Glossosomatidae	*Agapetusdelicatulus* McLachlan, 1884	INV05812	IBITR388-20	BOLD:AAE6313
		INV05813	IBITR389-20	
		INV05814	IBITR390-20	BOLD:AAE6313
	*Agapetusfuscipes* Curtis, 1834	INV05815	IBITR391-20	BOLD:AAJ7120
		INV05816	IBITR392-20	
		INV02468	IBITR099-20	BOLD:AEC9758
		INV05817	IBITR393-20	BOLD:AAJ7120
	*Agapetusincertulus* McLachlan, 1884	INV04759	IBITR273-20	BOLD:AEC9946
	*Agapetusnimbulus* McLachlan, 1879	INV04762	IBITR274-20	BOLD:AEM2297
	*Agapetusochripes* Curtis, 1834	INV04763	IBITR275-20	BOLD:AAB3823
	*Agapetussegovicus* Schmid, 1952	INV05818	IBITR394-20	BOLD:AEC7102
		INV05819	IBITR395-20	
		INV01163	IBITR075-20	BOLD:AEC7102
		INV04819	IBITR317-20	
	*Agapetustheischingeri* Malicky, 1980	INV05823	IBITR396-20	BOLD:AEL9298
	*Catagapetusmaclachlani* Malicky, 1975	INV05826	IBITR399-20	BOLD:ABA7173
		INV02477	IBITR108-20	
		INV02929	IBITR127-20	
		INV03936	IBITR169-20	
		INV05824	IBITR397-20	
		INV05825	IBITR398-20	
	*Glossosomaprivatum* McLachlan, 1884	INV05831	IBITR401-20	BOLD:AAM0930
		INV00461	IBITR050-20	
		INV00468	IBITR052-20	
		INV04688	IBITR219-20	
		INV04689	IBITR220-20	
		INV05830	IBITR400-20	
	*Synagapetusdiversus* (McLachlan, 1884)	INV05491	IBITR351-20	BOLD:ABX9025
	*Synagapetuslusitanicus* Malicky, 1980	INV05833	IBITR402-20	BOLD:AAO4326
Goeridae	*Larcasiapartita* Navás, 1917	INV00320	IBITR027-20	BOLD:AEC6981
		INV00327	IBITR029-20	
		INV00329	IBITR030-20	
		INV00341	IBITR034-20	
		INV00451	IBITR047-20	
		INV02473	IBITR104-20	
		INV02474	IBITR105-20	
		INV04733	IBITR254-20	
	*Silograellsii* Pictet, 1865	INV05951	IBITR413-20	BOLD:AEC7954
Helicopsychidae	*Helicopsychelusitanica* McLachlan, 1884	INV00005	IBITR012-20	BOLD:AEC8414
		INV04823	IBITR321-20	BOLD:AED0915
	*Helicopsyche* sp.	INV04824	IBITR322-20	BOLD:AEC8747
		INV04825	IBITR323-20	
Hydropsychidae	*Cheumatopsychelepida* (Pictet, 1834)	INV04778	IBITR283-20	BOLD:AAD1893
		INV04779	IBITR284-20	
		INV06149	IBITR432-20	
		INV06590	IBITR436-20	
	*Diplectronafelix* McLachlan, 1878	INV04718	IBITR243-20	BOLD:AAO2443
		INV04719	IBITR244-20	
		INV05479	IBITR339-20	
		INV05957	IBITR418-20	
	*Hydropsycheambigua* Schmid, 1973	INV03635	IBITR151-20	BOLD:AAB5092
		INV05480	IBITR340-20	BOLD:AAB9587
		INV05960	IBITR420-20	
		INV04720	IBITR245-20	
		INV04721	IBITR246-20	
		INV05493	IBITR353-20	
	*Hydropsychebrevis* Mosely, 1930	INV04780	IBITR285-20	BOLD:AEC9027
		INV04781	IBITR286-20	
	*Hydropsychebulbifera* McLachlan, 1878	INV04783	IBITR288-20	BOLD:AAO1831
		INV00809	IBITR071-20	
		INV04503	IBITR190-20	
		INV04782	IBITR287-20	
	*Hydropsychedinarica* Marinkovic-Gospodnetic, 1979	INV05956	IBITR417-20	BOLD:AAE5138
	*Hydropsycheexocellata* Dufour, 1841	INV02678	IBITR111-20	BOLD:AAF0933
		INV04785	IBITR289-20	
		INV00433	IBITR045-20	
		INV00434	IBITR046-20	
		INV02920	IBITR121-20	
		INV02922	IBITR123-20	
		INV02979	IBITR135-20	
	*Hydropsycheiberomaroccana* González & Malicky, 1999	INV04788	IBITR292-20	BOLD:AED0538
	*Hydropsycheinfernalis* Schmid, 1952	INV04789	IBITR293-20	BOLD:AAB5092
	*Hydropsycheinstabilis* (Curtis, 1834)	INV04722	IBITR247-20	BOLD:AAB1966
		INV04723	IBITR248-20	BOLD:ABZ1867
		INV05959	IBITR419-20	BOLD:AAB1966
	*Hydropsychelobata* McLachlan, 1884	INV04501	IBITR189-20	BOLD:AEC7586
		INV04787	IBITR291-20	
		INV00561	IBITR069-20	
		INV02669	IBITR110-20	
		INV03591	IBITR144-20	
		INV03592	IBITR145-20	
		INV04786	IBITR290-20	
	*Hydropsychepictetorum* Botosaneanu & Schmid, 1973	INV00421	IBITR037-20	BOLD:AAO2260
		INV02962	IBITR132-20	BOLD:AAB5092
		INV04790	IBITR294-20	BOLD:AAO2260
		INV05505	IBITR363-20	
	*Hydropsychesiltalai* Doehler, 1963	INV04269	IBITR175-20	BOLD:AAB5092
		INV00186	IBITR025-20	
		INV00460	IBITR049-20	
		INV04724	IBITR249-20	BOLD:AAB9587
		INV03680	IBITR160-20	BOLD:AAB5092
		INV05481	IBITR341-20	
		INV05482	IBITR342-20	
		INV05494	IBITR354-20	
	*Hydropsychetenuis* Navás, 1932	INV00318	IBITR026-20	BOLD:AAB9587
	*Hydropsychetibialis* McLachlan, 1884	INV04725	IBITR250-20	BOLD:AED0962
		INV04726	IBITR251-20	
		INV06211	IBITR433-20	
Hydroptilidae	*Agrayleasexmaculata* Curtis, 1834	INV04524	IBITR192-20	BOLD:AAE7232
		INV02924	IBITR124-20	
		INV02927	IBITR125-20	
		INV02928	IBITR126-20	
		INV03549	IBITR142-20	
	*Hydroptilafuentaldeala* Schmid, 1952	INV05477	IBITR337-20	BOLD:AEC8395
	*Ithytrichiaclavata* Morton, 1905	INV00520	IBITR066-20	BOLD:AEC8346
		INV00839	IBITR073-20	
	*Oxyethirafrici* Klapalek, 1891	INV05503	IBITR361-20	BOLD:ABY2898
Lepidostomatidae	*Lepidostomahirtum* (Fabricius, 1775)	INV00009	IBITR002-16	BOLD:AAB4052
		INV00010	IBITR003-16	
		INV00011	IBITR004-16	
		INV04579	IBITR202-20	
		INV04584	IBITR204-20	
Leptoceridae	*Adicellameridionalis* Morton, 1906	INV05510	IBITR367-20	BOLD:AEM0162
	*Adicellareducta* (McLachlan, 1865)	INV00422	IBITR038-20	BOLD:AAJ1835
		INV00426	IBITR041-20	
		INV00012	IBITR005-16	
		INV00013	IBITR006-16	
		INV00014	IBITR007-16	
		INV00470	IBITR053-20	
		INV00482	IBITR058-20	
		INV02475	IBITR106-20	
		INV04856	IBITR325-20	
	*Athripsodesalentexanus* Martín, González & Martínez, 2016	INV06592	IBITR437-20	BOLD:AAI7978
		INV06593	IBITR438-20	
	*Athripsodesbraueri* (Pictet, 1865)	INV04268	IBITR174-20	
		INV05485	IBITR345-20	
	*Athripsodesinaequalis* (McLachlan, 1884)	INV02463	IBITR094-20	BOLD:AED0841
		INV02919	IBITR120-20	
		INV03273	IBITR137-20	
	*Athripsodestavaresi* (Navás, 1916)	INV02764	IBITR116-20	BOLD:AEC8026
		INV03612	IBITR147-20	
		INV04754	IBITR268-20	
	*Ceracleaalbimacula* (Rambur, 1842)	INV00184	IBITR024-20	BOLD:AAN2950
		INV02233	IBITR084-20	
		INV04556	IBITR197-20	BOLD:AAD8966
	*Ceracleasobradieli* (Navás, 1917)	INV02950	IBITR001-16	BOLD:AAD8965
		INV02948	IBITR128-20	
		INV04554	IBITR196-20	
		INV04510	IBITR191-20	
		INV05474	IBITR334-20	
		INV05484	IBITR344-20	
	*Leptocerustineiformis* Curtis, 1834	INV00812	IBITR072-20	BOLD:AAJ1160
		INV00846	IBITR074-20	
		INV04287	IBITR177-20	
	*Mystacidesazureus* (Linnaeus, 1761)	INV02239	IBITR085-20	BOLD:AAB1494
		INV03563	IBITR143-20	
		INV04818	IBITR316-20	
	*Oecetistestacea* (Curtis, 1834)	INV05473	IBITR333-20	BOLD:AAD7208
	*Setodesargentipunctellus* McLachlan, 1877	INV05352	IBITR327-20	BOLD:ACB2223
		INV05353	IBITR328-20	
		INV00549	IBITR068-20	
		INV04817	IBITR315-20	
	*Triaenodesochreellus* McLachlan, 1877	INV02467	IBITR098-20	BOLD:AAJ8708
Limnephilidae	*Allogamuslaureatus* (Navás, 1918)	INV02246	IBITR086-20	BOLD:AEC7060
	*Allogamusligonifer* (McLachlan, 1876)	INV00321	IBITR028-20	BOLD:AAO2353
		INV02462	IBITR093-20	
		INV02466	IBITR097-20	
		INV04748	IBITR264-20	
		INV03724	IBITR164-20	
		INV03727	IBITR167-20	
	*Allogamusmortoni* (Navás, 1907)	INV04793	IBITR297-20	BOLD:AAM3837
		INV04794	IBITR298-20	
		INV04795	IBITR299-20	
	*Annitellaesparraguera* (Schmid, 1952)	INV05963	IBITR422-20	BOLD:AAM4103
	*Chaetopteryxatlantica* Malicky, 1975	INV05965	IBITR424-20	BOLD:AEC7901
	*Drususberthelemyi* Sipahiler, 1992	INV05964	IBITR423-20	BOLD:ACO5446
	*Drususbolivari* (McLachlan, 1880)	INV04791	IBITR295-20	BOLD:ACO5618
		INV04792	IBITR296-20	
	*Enoicylapusilla* (Burmeister, 1839)	INV04796	IBITR300-20	BOLD:AAO2902
	*Grammotauliussubmaculatus* (Rambur, 1842)	INV02799	IBITR117-20	BOLD:AEC8384
		INV04740	IBITR257-20	
	*Halesusradiatus* (Curtis, 1834)	INV01836	IBITR083-20	BOLD:AAF7718
		INV02469	IBITR100-20	
		INV04743	IBITR260-20	
		INV03722	IBITR162-20	
	*Limnephilusbipunctatus* Curtis, 1834	INV02609	IBITR109-20	BOLD:AAA4844
	*Limnephilusguadarramicus* Schmid, 1955	INV03661	IBITR156-20	BOLD:AEC8200
		INV03664	IBITR159-20	
		INV03946	IBITR170-20	
	*Limnephilushirsutus* (Pictet, 1834)	INV03655	IBITR155-20	BOLD:AAE6322
		INV03685	IBITR161-20	
		INV01281	IBITR078-20	
	*Limnephilussparsus* Curtis, 1834	INV04258	IBITR171-20	BOLD:AAB6375
		INV01284	IBITR079-20	
		INV03651	IBITR153-20	
		INV03653	IBITR154-20	
		INV04738	IBITR255-20	
	*Limnephilusvittatus* (Fabricius, 1798)	INV02256	IBITR089-20	BOLD:AAK8602
		INV04739	IBITR256-20	
		INV05478	IBITR338-20	
	*Mesophylaxaspersus* (Rambur, 1842)	INV04573	IBITR199-20	BOLD:AAG5761
		INV04662	IBITR207-20	
		INV04672	IBITR208-20	
		INV04530	IBITR193-20	
	*Potamophylaxcingulatus* (Stephens, 1837)	INV01300	IBITR081-20	BOLD:AAC4985
		INV04746	IBITR263-20	
		INV03662	IBITR157-20	
		INV05388	IBITR329-20	
		INV01299	IBITR080-20	BOLD:ABU7930
		INV02247	IBITR087-20	
		INV02253	IBITR088-20	
	*Potamophylaxlatipennis* (Curtis, 1834)	INV02257	IBITR090-20	
		INV02472	IBITR103-20	
		INV02800	IBITR118-20	
		INV04741	IBITR258-20	
	*Stenophylaxfissus* (McLachlan, 1875)	INV03616	IBITR148-20	BOLD:AEC6836
	*Stenophylaxmucronatus* McLachlan, 1880	INV03624	IBITR149-20	
		INV03642	IBITR152-20	
		INV04742	IBITR259-20	
		INV04744	IBITR261-20	BOLD:ABY2452
		INV02900	IBITR119-20	BOLD:AED0879
	*Stenophylaxpermistus* McLachlan, 1895	INV02951	IBITR130-20	
	*Stenophylaxsequax* (McLachlan, 1875)	INV02964	IBITR133-20	
		INV04655	IBITR205-20	
		INV04656	IBITR206-20	
		INV04745	IBITR262-20	BOLD:AAI0072
	*Stenophylaxvibex* (Curtis, 1834)	INV02957	IBITR131-20	BOLD:AAE8973
Odontoceridae	*Odontocerumalbicorne* (Scopoli, 1763)	INV00020	IBITR013-20	BOLD:AAB5626
		INV00021	IBITR008-16	
		INV05968	IBITR426-20	
		INV05970	IBITR427-20	
		INV05971	IBITR428-20	
		INV05972	IBITR429-20	
	*Odontocerumlusitanicum* Malicky, 1975	INV05508	IBITR365-20	BOLD:AEC9755
		INV05501	IBITR359-20	
Philopotamidae	*Chimarramarginata* (Linnaeus, 1767)	INV02459	IBITR091-20	BOLD:AAO1593
		INV00417	IBITR035-20	
		INV00419	IBITR036-20	
		INV00424	IBITR039-20	
		INV00425	IBITR040-20	
		INV00431	IBITR043-20	
		INV00432	IBITR044-20	
		INV00486	IBITR060-20	
		INV00506	IBITR064-20	
		INV02461	IBITR092-20	
		INV03259	IBITR136-20	
		INV05457	IBITR331-20	
	*Philopotamusamphilectus* McLachlan, 1884	INV04696	IBITR225-20	BOLD:AED0394
	*Philopotamusmontanuscaurelensis* González & Terra, 1979	INV02471	IBITR102-20	BOLD:AAO1570
		INV00334	IBITR032-20	
		INV00336	IBITR033-20	
		INV02465	IBITR096-20	
		INV02476	IBITR107-20	
		INV04694	IBITR223-20	BOLD:AEC7824
		INV04695	IBITR224-20	
	*Philopotamusperversus* McLachlan, 1884	INV00022	IBITR014-20	BOLD:AAO1569
		INV00023	IBITR015-20	
		INV00024	IBITR016-20	
		INV00025	IBITR017-20	
		INV05487	IBITR347-20	
		INV05835	IBITR403-20	
	*Philopotamusvariegatus* (Scopoli, 1763)	INV00458	IBITR048-20	BOLD:AEC7364
		INV00465	IBITR051-20	
	*Wormaldiabeaumonti* Schmid, 1952	INV01598	IBITR082-20	BOLD:AAO2217
		INV03725	IBITR165-20	BOLD:AAO2216
	*Wormaldiacorvina* (McLachlan, 1884)	INV04698	IBITR226-20	BOLD:ABU5927
		INV04699	IBITR227-20	
		INV05840	IBITR408-20	
		INV05841	IBITR409-20	
	*Wormaldialusitanica* González & Botosaneanu, 1983	INV05836	IBITR404-20	BOLD:AAO2217
	*Wormaldiaoccipitalis* (Pictet, 1834)	INV05838	IBITR406-20	BOLD:AED0699
		INV05837	IBITR405-20	
		INV05839	IBITR407-20	
	*Wormaldiatriangulifera* McLachlan, 1878	INV04765	IBITR276-20	BOLD:AAH9306
	*Wormaldiavariegata* mattheyi Schmid, 1952	INV04703	IBITR229-20	BOLD:AED0151
		INV04702	IBITR228-20	
		INV03723	IBITR163-20	
		INV03726	IBITR166-20	
Phryganeidae	*Agrypniavaria* (Fabricius, 1793)	INV03663	IBITR158-20	BOLD:AAE4334
		INV05340	IBITR326-20	
Polycentropodidae	*Cyrnuscintranus* McLachlan, 1884	INV05500	IBITR358-20	
	*Plectrocnemiageniculata* McLachlan, 1871	INV05953	IBITR415-20	
	*Plectrocnemialaetabilis* McLachlan, 1880	INV04295	IBITR184-20	BOLD:AAL4393
		INV02464	IBITR095-20	
		INV04704	IBITR230-20	
		INV04705	IBITR231-20	
		INV05389	IBITR330-20	
	*Polycentropuscorniger* McLachlan, 1884	INV04773	IBITR278-20	BOLD:AAL0051
		INV04772	IBITR277-20	
		INV05475	IBITR335-20	
		INV05483	IBITR343-20	
	*Polycentropusflavomaculatus* (Pictet, 1834)	INV00503	IBITR062-20	BOLD:AAC0971
		INV00504	IBITR063-20	BOLD:ACR2507
	*Polycentropusintricatus* Morton, 1910	INV04706	IBITR232-20	BOLD:AAL0054
		INV04707	IBITR233-20	
		INV05489	IBITR349-20	
	*Polycentropuskingi* McLachlan, 1881	INV04709	IBITR235-20	BOLD:AAL0060
		INV05498	IBITR357-20	
		INV00478	IBITR057-20	
		INV04708	IBITR234-20	
	*Polycentropustelifer* McLachlan, 1884	INV04417	IBITR185-20	BOLD:AAM0001
Psychomyiidae	*Lypeauripilis* McLachlan, 1884	INV04713	IBITR238-20	BOLD:AAO2229
	*Lypephaeopa* (Stephens, 1836)	INV00485	IBITR059-20	BOLD:AAC4581
	*Paduniellavandeli* Decamps, 1965	INV04774	IBITR279-20	BOLD:AAK7667
	*Psychomyiafragilis* (Pictet, 1834)	INV04775	IBITR280-20	
		INV02695	IBITR112-20	BOLD:AEC7914
	*Psychomyiapusilla* (Fabricius, 1781)	INV04710	IBITR236-20	BOLD:AAO1607
		INV00427	IBITR042-20	BOLD:AEC8086
		INV00806	IBITR070-20	
		INV02749	IBITR115-20	BOLD:AAO1607
		INV04711	IBITR237-20	
		INV05490	IBITR350-20	
		INV05495	IBITR355-20	BOLD:AEC8086
	*Tinodesassimilis* McLachlan, 1865	INV00521	IBITR067-20	BOLD:AAF7459
		INV01260	IBITR076-20	
		INV02921	IBITR122-20	
		INV04716	IBITR241-20	
		INV04717	IBITR242-20	
	*Tinodesfoedellus* McLachlan, 1884	INV04714	IBITR239-20	BOLD:AAL9978
		INV04715	IBITR240-20	
	*Tinodesmaculicornis* (Pictet, 1834)	INV05954	IBITR416-20	BOLD:AAF7446
	*Tinodeswaeneri* (Linnaeus, 1758)	INV04777	IBITR282-20	BOLD:AAB9068
		INV00491	IBITR061-20	
		INV01280	IBITR077-20	
		INV04576	IBITR200-20	
		INV04581	IBITR203-20	
		INV04423	IBITR187-20	
		INV04500	IBITR188-20	
		INV04776	IBITR281-20	
Ptilocolepidae	*Ptilocolepusextensus* McLachlan, 1884	INV04266	IBITR172-20	
		INV04690	IBITR221-20	BOLD:AAL2306
		INV04691	IBITR222-20	
	*Ptilocolepusgranulatus* (Pictet, 1834)	INV05967	IBITR425-20	
Rhyacophilidae	*Rhyacophilaadjuncta* McLachlan, 1884	INV00035	IBITR018-20	BOLD:AAD5575
		INV00330	IBITR031-20	
		INV04677	IBITR209-20	
		INV04678	IBITR210-20	
	*Rhyacophiladorsalis* (Curtis, 1834)	INV04811	IBITR314-20	BOLD:AAC4103
		INV05793	IBITR371-20	
		INV05794	IBITR372-20	
		INV05795	IBITR373-20	
	Rhyacophiladorsalisalbarracina Malicky, 2002	INV05790	IBITR368-20	
		INV05791	IBITR369-20	
	*Rhyacophilaevoluta* McLachlan, 1879	INV04807	IBITR310-20	BOLD:AAX8713
	*Rhyacophilaintermedia* McLachlan, 1868	INV04680	IBITR211-20	
		INV05507	IBITR364-20	
		INV04810	IBITR313-20	BOLD:AAF7929
	*Rhyacophilalaevis* Pictet, 1834	INV04809	IBITR312-20	BOLD:AAF8011
	*Rhyacophilalaufferi* Navás, 1918	INV05800	IBITR378-20	
	*Rhyacophilalusitanica* McLachlan, 1884	INV02967	IBITR134-20	BOLD:AEC8059
		INV00039	IBITR019-20	
		INV03379	IBITR138-20	
		INV03633	IBITR150-20	
		INV05504	IBITR362-20	
	*Rhyacophilamartynovi* Mosely, 1930	INV05799	IBITR377-20	
		INV04808	IBITR311-20	BOLD:AEC7148
	*Rhyacophilamelpomene* Malicky, 1976	INV04681	IBITR212-20	BOLD:AEM0544
		INV04682	IBITR213-20	
	*Rhyacophilameridionalis* Pictet, 1865	INV04683	IBITR214-20	BOLD:AEC9268
		INV04684	IBITR215-20	
	*Rhyacophilamocsaryitredosensis* Schmid, 1952	INV05796	IBITR374-20	BOLD:AEC7310
	*Rhyacophilamunda* McLachlan, 1862	INV05803	IBITR379-20	BOLD:AAM4449
		INV05804	IBITR380-20	
		INV05805	IBITR381-20	
		INV02949	IBITR129-20	BOLD:AEC7678
		INV03535	IBITR139-20	
		INV04572	IBITR198-20	BOLD:AAM4448
		INV04577	IBITR201-20	BOLD:AEC7678
		INV03900	IBITR168-20	
		INV04420	IBITR186-20	
	*Rhyacophilanevada* Schmid, 1952	INV05792	IBITR370-20	
	*Rhyacophilaobelix* Malicky, 1979	INV00044	IBITR020-20	BOLD:AEC8711
		INV00045	IBITR021-20	BOLD:AEC8521
		INV05947	IBITR410-20	BOLD:AEC8711
		INV05949	IBITR411-20	
	*Rhyacophilaobliterata* McLachlan, 1863	INV05797	IBITR375-20	
		INV05798	IBITR376-20	
	*Rhyacophilaoccidentalis* McLachlan, 1879	INV04685	IBITR216-20	BOLD:AAJ3548
		INV04686	IBITR217-20	
	*Rhyacophilapascoei* McLachlan, 1879	INV04755	IBITR269-20	BOLD:AEC7530
	*Rhyacophilapulchra* Schmid, 1952	INV04687	IBITR218-20	BOLD:AEC8544
	*Rhyacophilarelicta* McLachlan, 1879	INV05806	IBITR382-20	BOLD:AAI0887
		INV04532	IBITR195-20	
		INV04531	IBITR194-20	
		INV05807	IBITR383-20	
		INV06215	IBITR434-20	
	*Rhyacophilasociata* Navás 1916	INV05808	IBITR384-20	BOLD:AAD5575
		INV05809	IBITR385-20	
	*Rhyacophilaterpsichore* Malicky, 1976	INV04756	IBITR270-20	BOLD:AEC8427
	*Rhyacophilaterrai* González & Martínez, 2010	INV04757	IBITR271-20	BOLD:AEM3903
		INV04758	IBITR272-20	
	*Rhyacophilatristis* Pictet, 1834	INV05810	IBITR386-20	BOLD:ABA2486
		INV05811	IBITR387-20	
Sericostomatidae	*Schizopelexfestiva* (Rambur, 1842)	INV00053	IBITR022-20	BOLD:AAI0810
		INV00054	IBITR023-20	
		INV00513	IBITR065-20	
	*Sericostomapyrenaicum* Pictet, 1865	INV02747	IBITR113-20	BOLD:AAJ7690
		INV02748	IBITR114-20	
		INV04292	IBITR182-20	
		INV04749	IBITR265-20	
		INV04803	IBITR306-20	
		INV05509	IBITR366-20	BOLD:AEC8551
		INV04288	IBITR178-20	
		INV04289	IBITR179-20	BOLD:AAJ7690
		INV04290	IBITR180-20	BOLD:AEC8551
		INV04291	IBITR181-20	
		INV04293	IBITR183-20	BOLD:ABZ0751
		INV04804	IBITR307-20	BOLD:AAJ7690
		INV04805	IBITR308-20	
		INV04806	IBITR309-20	
		INV05472	IBITR332-20	BOLD:AEC8551
	*Sericostomavittatum* Rambur, 1842	INV04270	IBITR176-20	BOLD:ABZ0751
		INV04797	IBITR301-20	BOLD:AAM4952
		INV04822	IBITR320-20	BOLD:AAJ7690
		INV05486	IBITR346-20	BOLD:ABZ0751
		INV05492	IBITR352-20	BOLD:AAJ7690
		INV04752	IBITR266-20	
		INV04798	IBITR302-20	BOLD:AAM4952
		INV04799	IBITR303-20	
		INV04800	IBITR304-20	BOLD:AEC9666
		INV04801	IBITR305-20	BOLD:AAJ7690
		INV04820	IBITR318-20	
		INV04821	IBITR319-20	
Thremmatidae	*Thremmagallicum* McLachlan, 1880	INV05950	IBITR412-20	BOLD:AAF7946
		INV00056	IBITR009-16	
		INV00057	IBITR010-16	
		INV00058	IBITR011-16	
	*Thremmatellae* González, 1978	INV04833	IBITR324-20	BOLD:AAL9956
		INV05497	IBITR356-20	
